# Supplementary material for: Prevalence and anatomical significance of the persistent median artery: A cadaveric study
Source: PLoS One. 2025 Mar 31;20(3):e0320288. doi: 10.1371/journal.pone.0320288 (PMC11957254; doi:10.1371/journal.pone.0320288)
Supplement: S1 Table — (DOCX) [file pone.0320288.s001.docx]

Supplemental Table 1 Arterial diameter measurements in the presence or absence of a persistent median artery (PMA) and its subtypes.

| **Artery** | **Both limbs with either PMA** | **Both limbs without either PMA** | **Significance \|t\|** |
| --- | --- | --- | --- |
| Radial | 3.09 ± 0.56 mm (*n* = 34) | 3.13 ± 0.57 mm (*n* = 52) | *p* = 0.77 |
| Ulnar | 2.86 ± 0.65 mm (*n* = 36) | 2.75 ± 0.53 mm (*n* = 53) | *p* = 0.43 |
| SPA | 2.16 ± 0.65 mm (*n* = 31) | 2.02 ± 0.59 mm (*n* = 50) | *p* = 0.32* |
| DPA | 1.57 ± 0.50 mm (*n* = 35) | 1.49 ± 0.46 mm (*n* = 53) | *p* = 0.44 |
|  | **Both limbs with antebrachial-type PMA** | **Both limbs without antebrachial-type PMA** | **Significance** |
| Radial | 3.10 ± 0.55 mm (*n* = 22) | 3.12 ± 0.57 mm (*n* = 64) | *p* = 0.89 |
| Ulnar | 2.77 ± 0.67 mm (*n* = 23) | 2.81 ± 0.55 mm (*n* = 66) | *p* = 0.78 |
| SPA | 2.01 ± 0.59 mm (*n* = 21) | 2.09 ± 0.62 mm (*n* = 60) | *p* = 0.60 |
| DPA | 1.48 ± 0.41 mm (*n* = 23) | 1.54 ± 0.50 mm (*n* = 65) | *p* = 0.54 |
|  | **Left limb with either PMA** | **Left limb without either PMA** | **Significance** |
| Radial | 3.07 ± 0.62 mm (*n* = 18) | 3.11 ± 0.48 mm (*n* = 26) | p = 0.85 |
| Ulnar | 2.86 ± 0.70 mm (*n* = 20) | 2.77 ± 0.59 mm (*n* = 27) | *p* = 0.66 |
| SPA | 2.09 ± 0.54 mm (*n* = 16) | 2.05 ± 0.62 mm (*n* = 26) | *p* = 0.85 |
| DPA | 1.67 ± 0.56 mm (*n* = 20 | 1.55 ± 0.43 mm (*n* = 27) | *p* = 0.42 |
|  | **Left limb with antebrachial-type PMA** | **Left limb without antebrachial-type PMA** | **Significance** |
| Radial | 3.07 ± 0.58 mm (*n* = 10) | 3.09 ± 0.53 mm (*n* = 33) | *p* = 0.91 |
| Ulnar | 2.72 ± 0.69 mm (*n* = 11) | 2.85 ± 0.63 mm (*n* = 35) | *p* = 0.58 |
| SPA | 1.94 ± 0.37 mm (*n* = 10) | 2.10 ± 0.64 mm (*n* = 32) | *p* = 0.32 |
| DPA | 1.55 ± 0.47 mm (*n* = 11) | 1.62 ± 0.51 mm (*n* = 35) | *p* = 0.67 |
|  | **Right limb with either PMA** | **Right limb without either PMA** | **Significance** |
| Radial | 3.13 ± 0.50 mm (*n* = 17) | 3.15 ± 0.66 mm (*n* = 26) | *p* = 0.91 |
| Ulnar | 2.84 ± 0.60 mm (n = 17) | 2.74 ± 0.46 mm (*n* = 26) | *p* = 0.56 |
| SPA | 2.24 ± 0.76 mm (*n* = 15) | 1.98 ± 0.57 mm (*n* = 24) | *p* = 0.26 |
| DPA | 1.44 ± 0.36 mm (*n* = 16) | 1.44 ± 0.50 mm (*n* = 26) | *p* = 0.97 |
|  | **Right limb with antebrachial-type PMA** | **Right limb without antebrachial-type PMA** | **Significance** |
| Radial | 3.13 ± 0.54 mm (*n* = 5) | 3.15 ± 0.62 mm (*n* = 31) | *p* = 0.91 |
| Ulnar | 2.81 ± 0.67 mm (n = 12) | 2.76 ± 0.45 mm (*n* = 31) | *p* = 0.83 |
| SPA | 2.08 ± 0.76 mm (*n* = 11) | 2.08 ± 0.61 mm (*n* = 28) | *p* = 0.99 |
| DPA | 1.41 ± 0.37 mm (n = 12) | 1.45 ± 0.48 mm (*n* = 30) | *p* = 0.77 |

PMA: persistent median artery. SPA: superficial palmar arch. DPA: deep palmar arch. *indicates a single *tailed* t-test result is reported in the Results section.
