# Supplementary material for: Prevalence and anatomical significance of the persistent median artery: A cadaveric study
Source: PLoS One. 2025 Mar 31;20(3):e0320288. doi: 10.1371/journal.pone.0320288 (PMC11957254; doi:10.1371/journal.pone.0320288)
Supplement: S2 Table — (DOCX) [file pone.0320288.s002.docx]

Supplemental Table 2 Linear regression analysis demonstrates no correlation between persistent median artery (PMA) diameter and antebrachial artery diameter

|  | **Both Limbs** | | | | **Left Limb** | | | | **Right Limb** | | | |
| --- | --- | --- | --- | --- | --- | --- | --- | --- | --- | --- | --- | --- |
|  | Radial | Ulnar | SPA | DPA | Radial | Ulnar | SPA | DPA | Radial | Ulnar | SPA | DPA |
| Palmar-type | *r^2^* = 0.043 | *r^2^*= 0.02 | *r^2^*= 0.03 | *r^2^*= 0.11 | *r^2^*= 0.12 | *r^2^*= 0.05 | *r^2^*= 0.01 | *r^2^*= 0.09 | *r^2^*= 0.18 | *r^2^*= 0.64 | *r^2^*= 0.14 | *r^2^*= 0.44 |
| Antebrachial-type | *r^2^*= 0.036 | *r^2^*= 0.14 | *r^2^*= 0.03 | *r^2^*< 0.01 | *r^2^*= 0.04 | *r^2^*= 0.28 | *r^2^*= 0.02 | *r^2^*= 0.06 | *r^2^*< 0.01 | *r^2^*< 0.01 | *r^2^*= 0.31 | *r^2^*= 0.18 |
