# Supplementary material for: Prevalence and anatomical significance of the persistent median artery: A cadaveric study
Source: PLoS One. 2025 Mar 31;20(3):e0320288. doi: 10.1371/journal.pone.0320288 (PMC11957254; doi:10.1371/journal.pone.0320288)
Supplement: S3 Table — (DOCX) [file pone.0320288.s003.docx]

Supplemental Table 3 Raw data

| **Specimen** | **Laterality** | **Radial a** | **Radial in 1st metacarpal space** | **Ulnar a** | **SPA** | **DPA** | **Palm PMA** | **Ante PMA** |
| --- | --- | --- | --- | --- | --- | --- | --- | --- |
| 1 | L | 3.49 | 2.66 | 2.68 | 3.07 | 1.81 | n/a | n/a |
| 1 | L | 3.30 | 2.71 | 2.68 | 2.96 | 1.87 | n/a | n/a |
| 1 | L | 3.46 | 2.66 | 2.73 | 2.82 | 1.87 | n/a | n/a |
| 1 | R | 3.05 | 2.38 | 2.88 | 2.59 | 2.28 | n/a | n/a |
| 1 | R | 2.95 | 2.37 | 3.02 | 2.76 | 2.36 | n/a | n/a |
| 1 | R | 3.03 | 2.36 | 2.80 | 2.64 | 2.22 | n/a | n/a |
| 1 | Average L | 3.42 | 2.68 | 2.70 | 2.95 | 1.85 | n/a | n/a |
| 1 | Average R | 3.01 | 2.37 | 2.90 | 2.66 | 2.29 | n/a | n/a |
| 2 | L | 2.71 | 2.60 | 2.70 | 2.22 | 1.15 | n/a | n/a |
| 2 | L | 2.71 | 2.71 | 2.70 | 2.22 | 1.16 | n/a | n/a |
| 2 | L | 2.72 | 2.70 | 2.70 | 2.23 | 1.15 | n/a | n/a |
| 2 | R | 3.31 | 2.95 | 2.64 | 2.31 | 1.28 | n/a | n/a |
| 2 | R | 3.36 | 2.94 | 2.65 | 2.29 | 1.26 | n/a | n/a |
| 2 | R | 3.38 | 2.95 | 2.70 | 2.30 | 1.28 | n/a | n/a |
| 2 | Average L | 2.71 | 2.67 | 2.70 | 2.22 | 1.15 | n/a | n/a |
| 2 | Average R | 3.35 | 2.95 | 2.66 | 2.30 | 1.27 | n/a | n/a |
| 3 | L | 3.30 | 2.78 | 3.48 | 2.36 | 1.14 | n/a | 2.44 |
| 3 | L | 3.28 | 2.96 | 3.48 | 2.40 | 1.38 | n/a | 2.40 |
| 3 | L | 3.26 | 2.78 | 3.50 | 2.38 | 1.38 | n/a | 2.42 |
| 3 | R | n/a | n/a | n/a | n/a | n/a | n/a | n/a |
| 3 | R | n/a | n/a | n/a | n/a | n/a | n/a | n/a |
| 3 | R | n/a | n/a | n/a | n/a | n/a | n/a | n/a |
| 3 | Average L | 3.28 | 2.84 | 3.49 | 2.38 | 1.30 | n/a | 2.42 |
| 4 | L | 3.72 | 3.09 | 3.03 | 2.11 | 1.61 | n/a | n/a |
| 4 | L | 3.82 | 2.98 | 3.07 | 2.09 | 1.69 | n/a | n/a |
| 4 | L | 3.74 | 2.97 | 3.02 | 2.13 | 1.65 | n/a | n/a |
| 4 | R | 3.65 | 3.72 | 3.20 | 2.18 | 2.40 | n/a | n/a |
| 4 | R | 3.60 | 3.65 | 3.32 | 2.13 | 2.33 | n/a | n/a |
| 4 | R | 3.68 | 3.63 | 3.24 | 2.13 | 2.35 | n/a | n/a |
| 4 | Average L | 3.76 | 3.01 | 3.04 | 2.11 | 1.65 | n/a | n/a |
| 4 | Average R | 3.64 | 3.67 | 3.25 | 2.15 | 2.36 | n/a | n/a |
| 5 | L | 2.20 | 2.16 | 4.04 | 2.61 | 2.90 | 1.12 | 1.30 |
| 5 | L | 2.20 | 2.25 | 4.07 | 2.65 | 2.98 | 1.20 | 1.34 |
| 5 | L | 2.30 | 2.16 | 4.00 | 2.65 | 2.85 | 1.10 | 1.34 |
| 5 | R | 3.40 | 3.38 | 3.58 | 3.42 | 1.88 | 0.72 | 2.16 |
| 5 | R | 3.25 | 3.33 | 3.52 | 3.35 | 1.85 | 0.78 | 2.18 |
| 5 | R | 3.40 | 3.28 | 3.48 | 3.36 | 1.78 | 0.68 | 2.21 |
| 5 | Average L | 2.23 | 2.19 | 4.04 | 2.64 | 2.91 | 1.14 | 1.33 |
| 5 | Average R | 3.35 | 3.33 | 3.53 | 3.38 | 1.84 | 0.73 | 2.18 |
| 6 | L | 3.30 | 3.56 | 2.72 | 2.08 | 1.49 | n/a | n/a |
| 6 | L | 3.38 | 3.51 | 2.74 | 2.07 | 1.42 | n/a | n/a |
| 6 | L | 3.42 | 3.53 | 2.80 | 2.00 | 1.42 | n/a | n/a |
| 6 | R | n/a | n/a | n/a | n/a | n/a | n/a | n/a |
| 6 | R | n/a | n/a | n/a | n/a | n/a | n/a | n/a |
| 6 | R | n/a | n/a | n/a | n/a | n/a | n/a | n/a |
| 6 | Average L | 3.37 | 3.53 | 2.75 | 2.05 | 1.44 | n/a | n/a |
| 6 | Average R | n/a | n/a | n/a | n/a | n/a | n/a | n/a |
| 7 | L | n/a | 2.42 | 3.86 | n/a | 2.94 | 2.48 | 2.62 |
| 7 | L | n/a | 2.38 | 3.86 | n/a | 3.14 | 2.50 | 2.62 |
| 7 | L | n/a | 2.44 | 3.80 | n/a | 2.98 | 2.44 | 2.64 |
| 7 | R | n/a | n/a | n/a | n/a | n/a | n/a | n/a |
| 7 | R | n/a | n/a | n/a | n/a | n/a | n/a | n/a |
| 7 | R | n/a | n/a | n/a | n/a | n/a | n/a | n/a |
| 7 | Average L | n/a | 2.41 | 3.84 | n/a | 3.02 | 2.47 | 2.63 |
| 7 | Average R | n/a | n/a | n/a | n/a | n/a | n/a | n/a |
| 8 | L | 3.02 | 3.12 | 2.92 | n/a | 1.92 | n/a | n/a |
| 8 | L | 3.08 | 3.16 | 2.90 | n/a | 1.84 | n/a | n/a |
| 8 | L | 3.02 | 3.14 | 2.98 | n/a | 1.50 | n/a | n/a |
| 8 | R | 2.42 | 2.08 | 3.15 | n/a | 1.88 | n/a | n/a |
| 8 | R | 2.47 | 2.04 | 3.18 | n/a | 1.79 | n/a | n/a |
| 8 | R | 2.45 | 2.05 | 3.12 | n/a | 1.84 | n/a | n/a |
| 8 | Average L | 3.04 | 3.14 | 2.93 | n/a | 1.75 | n/a | n/a |
| 8 | Average R | 2.45 | 2.06 | 3.15 | n/a | 1.84 | n/a | n/a |
| 9 | L | 2.78 | 2.59 | 2.22 | 2.16 | 1.10 | n/a | n/a |
| 9 | L | 2.83 | 2.60 | 2.24 | 2.10 | 1.10 | n/a | n/a |
| 9 | L | 2.76 | 2.59 | 2.23 | 2.08 | 1.12 | n/a | n/a |
| 9 | R | 2.82 | 2.98 | 3.32 | 3.10 | 1.50 | n/a | n/a |
| 9 | R | 2.85 | 2.92 | 3.28 | 3.08 | 1.54 | n/a | n/a |
| 9 | R | 2.88 | 2.98 | 3.28 | 3.12 | 1.52 | n/a | n/a |
| 9 | Average L | 2.79 | 2.59 | 2.23 | 2.11 | 1.11 | n/a | n/a |
| 9 | Average R | 2.85 | 2.96 | 3.29 | 3.10 | 1.52 | n/a | n/a |
| 10 | L | 2.45 | 2.30 | 1.85 | 1.55 | 1.19 | 1.25 | 1.36 |
| 10 | L | 2.38 | 2.26 | 1.78 | 1.52 | 1.23 | 1.18 | 1.40 |
| 10 | L | 2.40 | 2.26 | 1.81 | 1.60 | 1.18 | 1.18 | 1.34 |
| 10 | R | 3.74 | 3.21 | 2.70 | 1.96 | 1.08 | 1.38 | 1.92 |
| 10 | R | 3.68 | 3.24 | 2.72 | 1.84 | 1.06 | 1.39 | 1.88 |
| 10 | R | 3.64 | 3.14 | 2.74 | 1.92 | 1.11 | 1.39 | 1.83 |
| 10 | Average L | 2.41 | 2.27 | 1.81 | 1.56 | 1.20 | 1.20 | 1.37 |
| 10 | Average R | 3.69 | 3.20 | 2.72 | 1.91 | 1.08 | 1.39 | 1.88 |
| 11 | L | 3.52 | 2.93 | 2.50 | 2.10 | 1.08 | n/a | n/a |
| 11 | L | 3.45 | 2.90 | 2.46 | 2.04 | 1.06 | n/a | n/a |
| 11 | L | 3.58 | 2.98 | 2.46 | 2.09 | 1.04 | n/a | n/a |
| 11 | R | 5.00 | 4.10 | 2.24 | 1.90 | 2.48 | n/a | n/a |
| 11 | R | 4.90 | 4.06 | 2.25 | 1.82 | 2.44 | n/a | n/a |
| 11 | R | 5.00 | 4.03 | 2.27 | 1.80 | 2.47 | n/a | n/a |
| 11 | Average L | 3.52 | 2.94 | 2.47 | 2.08 | 1.06 | n/a | n/a |
| 11 | Average R | 4.97 | 4.06 | 2.25 | 1.84 | 2.46 | n/a | n/a |
| 12 | L | 2.81 | 2.35 | 2.87 | 1.68 | 0.94 | n/a | 1.01 |
| 12 | L | 2.80 | 2.36 | 2.79 | 1.65 | 0.95 | n/a | 0.98 |
| 12 | L | 2.76 | 2.28 | 2.82 | 1.65 | 0.97 | n/a | 1.03 |
| 12 | R | 3.69 | 3.27 | 3.19 | 2.22 | 1.27 | n/a | n/a |
| 12 | R | 3.68 | 3.25 | 3.18 | 2.20 | 1.25 | n/a | n/a |
| 12 | R | 3.66 | 3.22 | 3.24 | 2.25 | 1.18 | n/a | n/a |
| 12 | Average L | 2.79 | 2.33 | 2.83 | 1.66 | 0.95 | n/a | 1.01 |
| 12 | Average R | 3.68 | 3.25 | 3.20 | 2.22 | 1.23 | n/a | n/a |
| 13 | L | 3.01 | 2.76 | 4.28 | n/a | 1.38 | n/a | 1.64 |
| 13 | L | 3.00 | 2.72 | 4.25 | n/a | 1.40 | n/a | 1.62 |
| 13 | L | 3.00 | 2.65 | 4.26 | n/a | 1.36 | n/a | 1.58 |
| 13 | R | 3.22 | 2.46 | 2.90 | 2.58 | 1.21 | n/a | 1.38 |
| 13 | R | 3.25 | 2.52 | 2.93 | 2.59 | 1.19 | n/a | 1.40 |
| 13 | R | 3.20 | 2.48 | 2.92 | 2.54 | 1.14 | n/a | 1.35 |
| 13 | Average L | 3.00 | 2.71 | 4.26 | n/a | 1.38 | n/a | 1.61 |
| 13 | Average R | 3.22 | 2.49 | 2.92 | 2.57 | 1.18 | n/a | 1.38 |
| 14 | L | 3.81 | 3.48 | 3.11 | 1.66 | 1.30 | n/a | 1.90 |
| 14 | L | 3.78 | 3.42 | 3.10 | 1.64 | 1.30 | n/a | 1.84 |
| 14 | L | 3.80 | 3.44 | 3.14 | 1.58 | 1.28 | n/a | 1.84 |
| 14 | R | 3.18 | 3.26 | 3.74 | 2.10 | 1.51 | n/a | n/a |
| 14 | R | 3.24 | 3.27 | 3.62 | 2.04 | 1.50 | n/a | n/a |
| 14 | R | 3.20 | 3.23 | 3.64 | 2.06 | 1.45 | n/a | n/a |
| 14 | Average L | 3.80 | 3.45 | 3.12 | 1.63 | 1.29 | n/a | 1.86 |
| 14 | Average R | 3.21 | 3.25 | 3.67 | 2.07 | 1.49 | n/a | n/a |
| 15 | L | 3.80 | 2.50 | 3.54 | 3.26 | 1.57 | 1.22 | 1.24 |
| 15 | L | 3.78 | 2.45 | 3.51 | 3.24 | 1.56 | 1.24 | 1.29 |
| 15 | L | 3.74 | 2.45 | 3.47 | 3.23 | 1.58 | 1.18 | 1.25 |
| 15 | R | 2.92 | 1.98 | 2.48 | n/a | 0.94 | n/a | n/a |
| 15 | R | 2.86 | 1.94 | 2.44 | n/a | 0.94 | n/a | n/a |
| 15 | R | 2.92 | 1.93 | 2.45 | n/a | 0.96 | n/a | n/a |
| 15 | Average L | 3.77 | 2.47 | 3.51 | 3.24 | 1.57 | 1.21 | 1.26 |
| 15 | Average R | 2.90 | 1.95 | 2.46 | n/a | 0.95 | n/a | n/a |
| 16 | L | 3.47 | 2.64 | 2.42 | n/a | 1.41 | n/a | 0.52 |
| 16 | L | 3.46 | 2.58 | 2.45 | n/a | 1.34 | n/a | 0.55 |
| 16 | L | 3.42 | 2.64 | 2.50 | n/a | 1.38 | n/a | 0.51 |
| 16 | R | n/a | n/a | n/a | n/a | n/a | n/a | n/a |
| 16 | R | n/a | n/a | n/a | n/a | n/a | n/a | n/a |
| 16 | R | n/a | n/a | n/a | n/a | n/a | n/a | n/a |
| 16 | Average L | 3.45 | 2.62 | 2.46 | n/a | 1.38 | n/a | 0.53 |
| 16 | Average R | n/a | n/a | n/a | n/a | n/a | n/a | n/a |
| 17 | L | 3.00 | 2.56 | 3.92 | 3.15 | 2.04 | n/a | n/a |
| 17 | L | 2.98 | 2.61 | 3.89 | 3.10 | 1.98 | n/a | n/a |
| 17 | L | 3.01 | 2.58 | 3.93 | 3.15 | 1.96 | n/a | n/a |
| 17 | R | 2.50 | 3.42 | 4.32 | 2.72 | 1.06 | n/a | 1.23 |
| 17 | R | 2.48 | 3.38 | 4.35 | 2.74 | 1.09 | n/a | 1.19 |
| 17 | R | 2.50 | 3.43 | 4.37 | 2.73 | 1.02 | n/a | 1.18 |
| 17 | Average L | 3.00 | 2.58 | 3.91 | 3.13 | 1.99 | n/a | n/a |
| 17 | Average R | 2.49 | 3.41 | 4.35 | 2.73 | 1.06 | n/a | 1.20 |
| 18 | L | n/a | n/a | n/a | n/a | n/a | n/a | n/a |
| 18 | L | n/a | n/a | n/a | n/a | n/a | n/a | n/a |
| 18 | L | n/a | n/a | n/a | n/a | n/a | n/a | n/a |
| 18 | R | n/a | n/a | n/a | n/a | n/a | n/a | n/a |
| 18 | R | n/a | n/a | n/a | n/a | n/a | n/a | n/a |
| 18 | R | n/a | n/a | n/a | n/a | n/a | n/a | n/a |
| 18 | Average L | n/a | n/a | n/a | n/a | n/a | n/a | n/a |
| 18 | Average R | n/a | n/a | n/a | n/a | n/a | n/a | n/a |
| 19 | L | 3.18 | 2.76 | 3.03 | 3.5 | 1.41 | n/a | n/a |
| 19 | L | 3.16 | 2.78 | 3.05 | 3.48 | 1.42 | n/a | n/a |
| 19 | L | 3.16 | 2.78 | 3.02 | 3.46 | 1.42 | n/a | n/a |
| 19 | R | n/a | n/a | n/a | n/a | n/a | n/a | n/a |
| 19 | R | n/a | n/a | n/a | n/a | n/a | n/a | n/a |
| 19 | R | n/a | n/a | n/a | n/a | n/a | n/a | n/a |
| 19 | Average L | 3.17 | 2.77 | 3.03 | 3.48 | 1.42 | n/a | n/a |
| 19 | Average R | n/a | n/a | n/a | n/a | n/a | n/a | n/a |
| 20 | L | 3.03 | 3.01 | 2.18 | 2.18 | 1.00 | n/a | n/a |
| 20 | L | 3.02 | 2.90 | 2.10 | 2.15 | 1.05 | n/a | n/a |
| 20 | L | 2.95 | 2.94 | 2.12 | 2.22 | 1.05 | n/a | n/a |
| 20 | R | 2.84 | 3.13 | 2.96 | 2.61 | 1.72 | n/a | 2.28 |
| 20 | R | 2.87 | 3.12 | 2.97 | 2.58 | 1.70 | n/a | 2.31 |
| 20 | R | 2.86 | 3.16 | 2.95 | 2.58 | 1.69 | n/a | 2.27 |
| 20 | Average L | 3.00 | 2.95 | 2.13 | 2.18 | 1.03 | n/a | n/a |
| 20 | Average R | 2.86 | 3.14 | 2.96 | 2.59 | 1.70 | n/a | 2.29 |
| 21 | L | 3.42 | 2.99 | 3.13 | 2.09 | 1.12 | n/a | n/a |
| 21 | L | 3.37 | 3.01 | 3.18 | 2.08 | 1.12 | n/a | n/a |
| 21 | L | 3.40 | 2.99 | 3.19 | 2.07 | 1.11 | n/a | n/a |
| 21 | R | n/a | n/a | n/a | n/a | n/a | n/a | n/a |
| 21 | R | n/a | n/a | n/a | n/a | n/a | n/a | n/a |
| 21 | R | n/a | n/a | n/a | n/a | n/a | n/a | n/a |
| 21 | Average L | 3.40 | 3.00 | 3.17 | 2.08 | 1.12 | n/a | n/a |
| 21 | Average R | n/a | n/a | n/a | n/a | n/a | n/a | n/a |
| 22 | L | 3.28 | 2.95 | 3.38 | n/a | 1.68 | 2.30 | 3.34 |
| 22 | L | 3.27 | 2.80 | 3.32 | n/a | 1.72 | 2.35 | 3.30 |
| 22 | L | 3.18 | 2.90 | 3.35 | n/a | 1.72 | 2.32 | 3.36 |
| 22 | R | 3.28 | 2.72 | 2.88 | n/a | 1.84 | 1.42 | 1.78 |
| 22 | R | 3.28 | 2.74 | 2.88 | n/a | 1.84 | 1.40 | 1.74 |
| 22 | R | 3.27 | 2.67 | 2.92 | n/a | 1.87 | 1.42 | 1.73 |
| 22 | Average L | 3.24 | 2.88 | 3.35 | n/a | 1.71 | 2.32 | 3.33 |
| 22 | Average R | 3.28 | 2.71 | 2.89 | n/a | 1.85 | 1.41 | 1.75 |
| 23 | L | 2.55 | 2.55 | 2.73 | 2.84 | 1.52 | 1.98 | 1.54 |
| 23 | L | 2.52 | 2.53 | 2.68 | 2.82 | 1.53 | 1.99 | 1.50 |
| 23 | L | 2.52 | 2.48 | 2.70 | 2.80 | 1.53 | 1.99 | 1.52 |
| 23 | R | 2.91 | 2.80 | 2.36 | 2.89 | n/a | 1.40 | 1.68 |
| 23 | R | 2.93 | 2.76 | 2.34 | 2.88 | n/a | 1.38 | 1.66 |
| 23 | R | 2.84 | 2.74 | 2.36 | 2.88 | n/a | 1.39 | 1.65 |
| 23 | Average L | 2.53 | 2.52 | 2.70 | 2.82 | 1.53 | 1.99 | 1.52 |
| 23 | Average R | 2.89 | 2.77 | 2.35 | 2.88 | n/a | 1.39 | 1.66 |
| 24 | L | 2.81 | 2.75 | 2.42 | 1.67 | 1.24 | n/a | 1.00 |
| 24 | L | 2.78 | 2.68 | 2.35 | 1.67 | 1.25 | n/a | 0.97 |
| 24 | L | 2.80 | 2.69 | 2.42 | 1.68 | 1.27 | n/a | 0.93 |
| 24 | R | 2.42 | 2.42 | 2.68 | 1.48 | 1.06 | n/a | n/a |
| 24 | R | 2.39 | 2.38 | 2.68 | 1.49 | 1.04 | n/a | n/a |
| 24 | R | 2.45 | 2.39 | 2.71 | 1.48 | 1.02 | n/a | n/a |
| 24 | Average L | 2.80 | 2.71 | 2.40 | 1.67 | 1.25 | n/a | 0.97 |
| 24 | Average R | 2.42 | 2.40 | 2.69 | 1.48 | 1.04 | n/a | n/a |
| 25 | L | n/a | 2.27 | 2.07 | 2.18 | 1.39 | n/a | 0.87 |
| 25 | L | n/a | 2.24 | 2.08 | 2.15 | 1.39 | n/a | 0.86 |
| 25 | L | n/a | 2.26 | 2.02 | 2.18 | 1.37 | n/a | 0.85 |
| 25 | R | 3.19 | 2.83 | 2.37 | 2.07 | 1.13 | n/a | n/a |
| 25 | R | 3.18 | 2.80 | 2.38 | 2.04 | 1.09 | n/a | n/a |
| 25 | R | 3.20 | 2.84 | 2.38 | 2.05 | 1.13 | n/a | n/a |
| 25 | Average L | n/a | 2.26 | 2.06 | 2.17 | 1.38 | n/a | 0.86 |
| 25 | Average R | 3.19 | 2.82 | 2.38 | 2.05 | 1.12 | n/a | n/a |
| 26 | L | n/a | n/a | n/a | n/a | n/a | n/a | n/a |
| 26 | L | n/a | n/a | n/a | n/a | n/a | n/a | n/a |
| 26 | L | n/a | n/a | n/a | n/a | n/a | n/a | n/a |
| 26 | R | 3.90 | 3.06 | 1.90 | n/a | 1.98 | n/a | 1.74 |
| 26 | R | 3.87 | 3.00 | 1.95 | n/a | 1.94 | n/a | 1.72 |
| 26 | R | 3.84 | 3.02 | 1.96 | n/a | 1.94 | n/a | 1.73 |
| 26 | Average L | n/a | n/a | n/a | n/a | n/a | n/a | n/a |
| 26 | Average R | 3.87 | 3.03 | 1.94 | n/a | 1.95 | n/a | 1.73 |
| 27 | L | n/a | n/a | n/a | n/a | n/a | n/a | n/a |
| 27 | L | n/a | n/a | n/a | n/a | n/a | n/a | n/a |
| 27 | L | n/a | n/a | n/a | n/a | n/a | n/a | n/a |
| 27 | R | 2.79 | 2.78 | 2.94 | 2.36 | 1.44 | n/a | 1.74 |
| 27 | R | 2.82 | 2.82 | 2.90 | 2.37 | 1.44 | n/a | 1.76 |
| 27 | R | 2.81 | 2.82 | 2.88 | 2.35 | 1.44 | n/a | 1.75 |
| 27 | Average L | n/a | n/a | n/a | n/a | n/a | n/a | n/a |
| 27 | Average R | 2.81 | 2.81 | 2.91 | 2.36 | 1.44 | n/a | 1.75 |
| 28 | L | n/a | n/a | n/a | n/a | n/a | n/a | n/a |
| 28 | L | n/a | n/a | n/a | n/a | n/a | n/a | n/a |
| 28 | L | n/a | n/a | n/a | n/a | n/a | n/a | n/a |
| 28 | R | 2.80 | 2.10 | 2.00 | 1.19 | 1.18 | n/a | n/a |
| 28 | R | 2.85 | 2.08 | 1.93 | 1.16 | 1.16 | n/a | n/a |
| 28 | R | 2.85 | 2.10 | 1.99 | 1.16 | 1.17 | n/a | n/a |
| 28 | Average L | n/a | n/a | n/a | n/a | n/a | n/a | n/a |
| 28 | Average R | 2.83 | 2.09 | 1.97 | 1.17 | 1.17 | n/a | n/a |
| 29 | L | 3.11 | 2.76 | 3.63 | 2.37 | 1.47 | n/a | n/a |
| 29 | L | 3.12 | 2.74 | 3.64 | 2.34 | 1.46 | n/a | n/a |
| 29 | L | 3.05 | 2.79 | 3.57 | 2.32 | 1.45 | n/a | n/a |
| 29 | R | 2.28 | 2.30 | 3.64 | 2.98 | 1.35 | n/a | 1.34 |
| 29 | R | 2.33 | 2.32 | 3.64 | 2.95 | 1.28 | n/a | 1.39 |
| 29 | R | 2.34 | 2.29 | 3.60 | 3.02 | 1.27 | n/a | 1.37 |
| 29 | avg. left | 3.09 | 2.76 | 3.61 | 2.34 | 1.46 | n/a | n/a |
| 29 | avg. right | 2.32 | 2.30 | 3.63 | 2.98 | 1.30 | n/a | 1.37 |
| 30 | L | n/a | n/a | 2.12 | 1.60 | 1.33 | n/a | n/a |
| 30 | L | n/a | n/a | 2.15 | 1.57 | 1.34 | n/a | n/a |
| 30 | L | n/a | n/a | 2.16 | 1.57 | 1.38 | n/a | n/a |
| 30 | R | 2.93 | 2.71 | 2.07 | 0.55 | 1.76 | n/a | 1.35 |
| 30 | R | 2.98 | 2.74 | 2.10 | 0.50 | 1.74 | n/a | 1.31 |
| 30 | R | 2.88 | 2.69 | 2.09 | 0.53 | 1.75 | n/a | 1.33 |
| 30 | avg. left | n/a | n/a | 2.14 | 1.58 | 1.35 | n/a | n/a |
| 30 | avg. right | 2.93 | 2.71 | 2.09 | 0.53 | 1.75 | n/a | 1.33 |
| 31 | L | 2.30 | 2.46 | 2.87 | 2.34 | 1.04 | n/a | n/a |
| 31 | L | 2.34 | 2.52 | 2.90 | 2.35 | 1.03 | n/a | n/a |
| 31 | L | 2.28 | 2.49 | 2.87 | 2.37 | 1.07 | n/a | n/a |
| 31 | R | 2.18 | 1.84 | 3.45 | 2.55 | 1.20 | n/a | n/a |
| 31 | R | 2.16 | 1.86 | 3.43 | 2.53 | 1.15 | n/a | n/a |
| 31 | R | 2.20 | 1.86 | 3.48 | 2.54 | 1.16 | n/a | n/a |
| 31 | avg. left | 2.31 | 2.49 | 2.88 | 2.35 | 1.05 | n/a | n/a |
| 31 | avg. right | 2.18 | 1.85 | 3.45 | 2.54 | 1.17 | n/a | n/a |
| 32 | L | 3.24 | 3.21 | 2.38 | 2.32 | 2.04 | n/a | 1.02 |
| 32 | L | 3.24 | 3.23 | 2.37 | 2.35 | 2.08 | n/a | 1.00 |
| 32 | L | 3.20 | 3.21 | 2.41 | 2.34 | 2.03 | n/a | 0.99 |
| 32 | R | 3.24 | 3.31 | 2.33 | 1.76 | 1.51 | n/a | 1.04 |
| 32 | R | 3.18 | 3.33 | 2.30 | 1.68 | 1.49 | n/a | 1.06 |
| 32 | R | 3.23 | 3.33 | 2.32 | 1.72 | 1.46 | n/a | 1.01 |
| 32 | avg. left | 3.23 | 3.22 | 2.39 | 2.34 | 2.05 | n/a | 1.00 |
| 32 | avg. right | 3.22 | 3.32 | 2.32 | 1.72 | 1.49 | n/a | 1.04 |
| 33 | L | 3.34 | 2.84 | 3.18 | 2.26 | 1.18 | n/a | n/a |
| 33 | L | 3.26 | 2.80 | 3.16 | 2.26 | 1.20 | n/a | n/a |
| 33 | L | 3.31 | 2.84 | 3.22 | 2.24 | 1.19 | n/a | n/a |
| 33 | R | 3.68 | 3.34 | 2.29 | 1.40 | 1.53 | n/a | n/a |
| 33 | R | 3.65 | 3.32 | 2.32 | 1.44 | 1.47 | n/a | n/a |
| 33 | R | 3.68 | 3.32 | 2.24 | 1.48 | 1.45 | n/a | n/a |
| 33 | avg. left | 3.30 | 2.83 | 3.19 | 2.25 | 1.19 | n/a | n/a |
| 33 | avg. right | 3.67 | 3.33 | 2.28 | 1.44 | 1.48 | n/a | n/a |
| 34 | L | 3.01 | 2.58 | 2.19 | 1.54 | 1.59 | n/a | n/a |
| 34 | L | 3.06 | 2.51 | 2.17 | 1.51 | 1.60 | n/a | n/a |
| 34 | L | 3.03 | 2.55 | 2.20 | 1.48 | 1.52 | n/a | n/a |
| 34 | R | 3.82 | 2.98 | 2.64 | 1.49 | 1.50 | n/a | n/a |
| 34 | R | 3.84 | 2.92 | 2.65 | 1.51 | 1.56 | n/a | n/a |
| 34 | R | 3.87 | 2.94 | 2.65 | 1.51 | 1.55 | n/a | n/a |
| 34 | avg. left | 3.03 | 2.55 | 2.19 | 1.51 | 1.57 | n/a | n/a |
| 34 | avg. right | 3.84 | 2.95 | 2.65 | 1.50 | 1.54 | n/a | n/a |
| 35 | L | 2.64 | 1.94 | 3.98 | 2.70 | 1.88 | n/a | n/a |
| 35 | L | 2.62 | 1.94 | 4.01 | 2.68 | 1.85 | n/a | n/a |
| 35 | L | 2.65 | 1.93 | 4.01 | 2.71 | 1.88 | n/a | n/a |
| 35 | R | 2.54 | 2.50 | 3.03 | 2.58 | 1.38 | 0.75 | 1.54 |
| 35 | R | 2.54 | 2.52 | 3.06 | 2.57 | 1.36 | 0.73 | 1.56 |
| 35 | R | 2.47 | 2.51 | 3.05 | 2.61 | 1.40 | 0.73 | 1.60 |
| 35 | avg. left | 2.64 | 1.94 | 4.00 | 2.70 | 1.87 | n/a | n/a |
| 35 | avg. right | 2.52 | 2.51 | 3.05 | 2.59 | 1.38 | 0.74 | 1.57 |
| 36 | L | 2.16 | 3.02 | 2.63 | 1.36 | 1.45 | n/a | 1.21 |
| 36 | L | 2.14 | 3.00 | 2.65 | 1.32 | 1.40 | n/a | 1.20 |
| 36 | L | 2.20 | 3.03 | 2.60 | 1.32 | 1.43 | n/a | 1.20 |
| 36 | R | 2.98 | 2.92 | 2.26 | 1.13 | 1.82 | n/a | 1.39 |
| 36 | R | 2.97 | 2.94 | 2.24 | 1.10 | 1.82 | n/a | 1.39 |
| 36 | R | 2.99 | 2.92 | 2.26 | 1.10 | 1.86 | n/a | 1.37 |
| 36 | avg. left | 2.17 | 3.02 | 2.63 | 1.33 | 1.43 | n/a | 1.20 |
| 36 | avg. right | 2.98 | 2.93 | 2.25 | 1.11 | 1.83 | n/a | 1.38 |
| 37 | L | 2.84 | 2.67 | 3.05 | 2.07 | 1.53 | n/a | n/a |
| 37 | L | 2.85 | 2.66 | 3.06 | 2.04 | 1.55 | n/a | n/a |
| 37 | L | 2.85 | 2.62 | 3.09 | 2.08 | 1.50 | n/a | n/a |
| 37 | R | 3.26 | 3.25 | 2.85 | 1.75 | 1.68 | n/a | n/a |
| 37 | R | 3.26 | 3.23 | 2.84 | 1.74 | 1.71 | n/a | n/a |
| 37 | R | 3.25 | 3.18 | 2.88 | 1.76 | 1.71 | n/a | n/a |
| 37 | avg. left | 2.85 | 2.65 | 3.07 | 2.06 | 1.53 | n/a | n/a |
| 37 | avg. right | 3.26 | 3.22 | 2.86 | 1.75 | 1.70 | n/a | n/a |
| 38 | L | 3.54 | 3.44 | 1.79 | 1.76 | 2.46 | n/a | n/a |
| 38 | L | 3.58 | 3.42 | 1.78 | 1.78 | 2.46 | n/a | n/a |
| 38 | L | 3.57 | 3.44 | 1.73 | 1.74 | 2.44 | n/a | n/a |
| 38 | R | 3.80 | 3.29 | 2.53 | 1.94 | 1.62 | n/a | n/a |
| 38 | R | 3.78 | 3.31 | 2.55 | 1.90 | 1.64 | n/a | n/a |
| 38 | R | 3.76 | 3.28 | 2.54 | 1.92 | 1.62 | n/a | n/a |
| 38 | avg. left | 3.56 | 3.43 | 1.77 | 1.76 | 2.45 | n/a | n/a |
| 38 | avg. right | 3.78 | 3.29 | 2.54 | 1.92 | 1.63 | n/a | n/a |
| 39 | L | 3.18 | 3.31 | 3.02 | 1.42 | 1.96 | n/a | n/a |
| 39 | L | 3.17 | 3.27 | 3.04 | 1.40 | 1.94 | n/a | n/a |
| 39 | L | 3.20 | 3.33 | 3.04 | 1.39 | 1.90 | n/a | n/a |
| 39 | R | 3.54 | 3.48 | 2.72 | 1.33 | 1.04 | n/a | n/a |
| 39 | R | 3.56 | 3.47 | 2.68 | 1.28 | 1.04 | n/a | n/a |
| 39 | R | 3.56 | 3.51 | 2.71 | 1.32 | 1.02 | n/a | n/a |
| 39 | avg. left | 3.18 | 3.30 | 3.03 | 1.40 | 1.93 | n/a | n/a |
| 39 | avg. right | 3.55 | 3.49 | 2.70 | 1.31 | 1.03 | n/a | n/a |
| 40 | L | 2.82 | 3.50 | 2.54 | 1.42 | 1.42 | 1.46 | 1.32 |
| 40 | L | 2.81 | 3.48 | 2.46 | 1.44 | 1.43 | 1.50 | 1.27 |
| 40 | L | 2.84 | 3.54 | 2.53 | 1.42 | 1.43 | 1.47 | 1.33 |
| 40 | R | 3.13 | 3.04 | 3.04 | 1.91 | 1.44 | n/a | n/a |
| 40 | R | 3.13 | 3.02 | 2.98 | 1.86 | 1.42 | n/a | n/a |
| 40 | R | 3.10 | 3.04 | 3.02 | 1.89 | 1.45 | n/a | n/a |
| 40 | avg. left | 2.82 | 3.51 | 2.51 | 1.43 | 1.43 | 1.48 | 1.31 |
| 40 | avg. right | 3.12 | 3.03 | 3.01 | 1.89 | 1.44 | n/a | n/a |
| 41 | L | 3.00 | 2.97 | 2.78 | 1.77 | 2.00 | n/a | n/a |
| 41 | L | 3.01 | 2.93 | 2.82 | 1.76 | 1.98 | n/a | n/a |
| 41 | L | 2.96 | 2.93 | 2.78 | 1.73 | 2.02 | n/a | n/a |
| 41 | R | 2.90 | 2.92 | 3.14 | 2.84 | 1.41 | n/a | n/a |
| 41 | R | 2.86 | 2.90 | 3.10 | 2.85 | 1.42 | n/a | n/a |
| 41 | R | 2.88 | 2.94 | 3.10 | 2.84 | 1.40 | n/a | n/a |
| 41 | avg. left | 2.99 | 2.94 | 2.79 | 1.75 | 2.00 | n/a | n/a |
| 41 | avg. right | 2.88 | 2.92 | 3.11 | 2.84 | 1.41 | n/a | n/a |
| 42 | L | 2.40 | 1.89 | 2.54 | 2.20 | 1.46 | n/a | 1.04 |
| 42 | L | 2.41 | 1.86 | 2.52 | 2.21 | 1.60 | n/a | 1.03 |
| 42 | L | 2.40 | 1.90 | 2.49 | 2.24 | 1.62 | n/a | 1.03 |
| 42 | R | 2.89 | 2.86 | 2.06 | 1.84 | 0.76 | n/a | n/a |
| 42 | R | 2.91 | 2.85 | 2.07 | 1.82 | 0.76 | n/a | n/a |
| 42 | R | 2.92 | 2.88 | 2.08 | 1.82 | 0.80 | n/a | n/a |
| 42 | avg. left | 2.40 | 1.88 | 2.52 | 2.22 | 1.56 | n/a | 1.03 |
| 42 | avg. right | 2.91 | 2.86 | 2.07 | 1.83 | 0.77 | n/a | n/a |
| 43 | L | 3.38 | 3.10 | 3.06 | 2.69 | 1.69 | n/a | n/a |
| 43 | L | 3.38 | 3.10 | 3.06 | 2.70 | 1.70 | n/a | n/a |
| 43 | L | 3.37 | 3.13 | 3.06 | 2.68 | 1.71 | n/a | n/a |
| 43 | R | 3.20 | 3.02 | 3.18 | 1.66 | 1.54 | n/a | n/a |
| 43 | R | 3.18 | 3.00 | 3.16 | 1.67 | 1.52 | n/a | n/a |
| 43 | R | 3.19 | 3.03 | 3.19 | 1.70 | 1.54 | n/a | n/a |
| 43 | avg. left | 3.38 | 3.11 | 3.06 | 2.69 | 1.70 | n/a | n/a |
| 43 | avg. right | 3.19 | 3.02 | 3.18 | 1.68 | 1.53 | n/a | n/a |
| 44 | L | 2.71 | 2.50 | 2.88 | 1.79 | 1.69 | n/a | n/a |
| 44 | L | 2.69 | 2.48 | 2.86 | 1.80 | 1.71 | n/a | n/a |
| 44 | L | 2.70 | 2.54 | 2.89 | 1.81 | 1.68 | n/a | n/a |
| 44 | R | 3.54 | 3.33 | 2.68 | 1.94 | 1.01 | n/a | 1.06 |
| 44 | R | 3.56 | 3.33 | 2.70 | 1.93 | 0.96 | n/a | 1.07 |
| 44 | R | 3.56 | 3.30 | 2.66 | 1.92 | 0.99 | n/a | 1.08 |
| 44 | avg. left | 2.70 | 2.51 | 2.88 | 1.80 | 1.69 | n/a | n/a |
| 44 | avg. right | 3.55 | 1.80 | 2.68 | 1.93 | 0.99 | n/a | 1.07 |
| 45 | L | 4.20 | 3.54 | 3.02 | 2.31 | 1.60 | 0.59 | 1.80 |
| 45 | L | 4.22 | 3.55 | 3.04 | 2.28 | 1.61 | 0.59 | 1.81 |
| 45 | L | 4.20 | 3.54 | 3.01 | 2.28 | 1.59 | 0.58 | 1.78 |
| 45 | R | 4.22 | 3.00 | 2.97 | 2.67 | 1.47 | n/a | 1.14 |
| 45 | R | 4.22 | 3.02 | 3.00 | 2.64 | 1.46 | n/a | 1.18 |
| 45 | R | 4.20 | 3.00 | 2.97 | 2.67 | 1.47 | n/a | 1.18 |
| 45 | avg. left | 4.21 | 3.54 | 3.02 | 2.29 | 1.60 | 0.59 | 1.80 |
| 45 | avg. right | 4.21 | 3.01 | 2.98 | 2.66 | 1.47 | n/a | 1.17 |
| 46 | L | 3.14 | 2.53 | 2.28 | 2.25 | 1.80 | n/a | 1.34 |
| 46 | L | 3.12 | 2.54 | 2.29 | 2.24 | 1.79 | n/a | 1.35 |
| 46 | L | 3.11 | 2.55 | 2.28 | 2.21 | 1.80 | n/a | 1.35 |
| 46 | R | n/a | n/a | n/a | n/a | n/a | n/a | 1.68 |
| 46 | R | n/a | n/a | n/a | n/a | n/a | n/a | 1.66 |
| 46 | R | n/a | n/a | n/a | n/a | n/a | n/a | 1.67 |
| 46 | avg. left | 3.12 | 2.54 | 2.28 | 2.23 | 1.80 | n/a | 1.35 |
| 46 | avg. right | n/a | n/a | n/a | n/a | n/a | n/a | 1.67 |
| 47 | L | 3.50 | 3.22 | 3.21 | 1.46 | 0.92 | n/a | n/a |
| 47 | L | 3.48 | 3.24 | 3.19 | 1.46 | 0.93 | n/a | n/a |
| 47 | L | 3.46 | 3.19 | 3.18 | 1.49 | 0.92 | n/a | n/a |
| 47 | R | 3.11 | 2.50 | 2.70 | 1.69 | 0.74 | n/a | 1.38 |
| 47 | R | 3.12 | 2.51 | 2.72 | 1.70 | 0.76 | n/a | 1.38 |
| 47 | R | 3.13 | 2.48 | 2.72 | 1.72 | 0.76 | n/a | 1.40 |
| 47 | avg. left | 3.48 | 3.22 | 3.19 | 1.47 | 0.92 | n/a | n/a |
| 47 | avg. right | 3.12 | 2.50 | 2.71 | 1.70 | 0.75 | n/a | 1.39 |
| 48 | L | 4.06 | 4.00 | 1.88 | 1.76 | 2.67 | n/a | 1.26 |
| 48 | L | 4.06 | 4.00 | 1.89 | 1.78 | 2.64 | n/a | 1.26 |
| 48 | L | 4.10 | 4.01 | 1.91 | 1.75 | 2.64 | n/a | 1.25 |
| 48 | R | 2.74 | 2.86 | 2.08 | 2.02 | 1.54 | n/a | n/a |
| 48 | R | 2.75 | 2.87 | 2.05 | 2.03 | 1.50 | n/a | n/a |
| 48 | R | 2.75 | 2.89 | 2.04 | 2.04 | 1.50 | n/a | n/a |
| 48 | avg. left | 4.07 | 4.00 | 1.89 | 1.76 | 2.65 | n/a | 1.26 |
| 48 | avg. right | 2.75 | 2.87 | 2.06 | 2.03 | 1.51 | n/a | n/a |
| 49 | L | 4.40 | 3.85 | 1.59 | 1.34 | 2.64 | n/a | n/a |
| 49 | L | 4.38 | 3.87 | 1.60 | 1.36 | 2.60 | n/a | n/a |
| 49 | L | 4.42 | 3.84 | 1.62 | 1.34 | 2.60 | n/a | n/a |
| 49 | R | 4.15 | 3.94 | 2.26 | 2.85 | 2.17 | n/a | n/a |
| 49 | R | 4.12 | 3.94 | 2.26 | 2.85 | 2.18 | n/a | n/a |
| 49 | R | 4.12 | 3.92 | 2.25 | 3.84 | 2.16 | n/a | n/a |
| 49 | avg. left | 4.40 | 3.85 | 1.60 | 1.35 | 2.61 | n/a | n/a |
| 49 | avg. right | 4.13 | 3.93 | 2.26 | 3.18 | 2.17 | n/a | n/a |
| 50 | L | 2.60 | 2.40 | 2.15 | 1.26 | 1.54 | n/a | n/a |
| 50 | L | 2.62 | 2.42 | 2.10 | 1.27 | 1.51 | n/a | n/a |
| 50 | L | 2.62 | 2.38 | 2.15 | 1.27 | 1.53 | n/a | n/a |
| 50 | R | 2.20 | 2.82 | 2.38 | 1.34 | 0.67 | n/a | n/a |
| 50 | R | 2.20 | 2.79 | 2.38 | 1.32 | 0.68 | n/a | n/a |
| 50 | R | 2.24 | 2.81 | 2.34 | 1.32 | 0.70 | n/a | n/a |
| 50 | avg. left | 2.61 | 2.40 | 2.13 | 1.27 | 1.53 | n/a | n/a |
| 50 | avg. right | 2.21 | 2.81 | 2.37 | 1.33 | 0.68 | n/a | n/a |
| 51 | L | 2.04 | 2.02 | 2.20 | 0.68 | 1.31 | n/a | n/a |
| 51 | L | 2.07 | 2.04 | 2.20 | 0.66 | 1.30 | n/a | n/a |
| 51 | L | 2.02 | 2.05 | 2.22 | 0.65 | 1.32 | n/a | n/a |
| 51 | R | 2.04 | 2.03 | 2.79 | 1.31 | 0.56 | n/a | n/a |
| 51 | R | 2.02 | 2.02 | 2.81 | 1.33 | 0.53 | n/a | n/a |
| 51 | R | 2.03 | 2.01 | 2.76 | 1.29 | 0.55 | n/a | n/a |
| 51 | avg. left | 2.04 | 2.04 | 2.21 | 0.66 | 1.31 | n/a | n/a |
| 51 | avg. right | 2.03 | 2.02 | 2.79 | 1.31 | 0.55 | n/a | n/a |
| 52 | L | n/a | n/a | n/a | n/a | n/a | n/a | n/a |
| 52 | L | n/a | n/a | n/a | n/a | n/a | n/a | n/a |
| 52 | L | n/a | n/a | n/a | n/a | n/a | n/a | n/a |
| 52 | R | n/a | n/a | n/a | n/a | n/a | n/a | n/a |
| 52 | R | n/a | n/a | n/a | n/a | n/a | n/a | n/a |
| 52 | R | n/a | n/a | n/a | n/a | n/a | n/a | n/a |
| 52 | avg. left | n/a | n/a | n/a | n/a | n/a | n/a | n/a |
| 52 | avg. right | n/a | n/a | n/a | n/a | n/a | n/a | n/a |
| 53 | L | n/a | n/a | n/a | n/a | n/a | n/a | n/a |
| 53 | L | n/a | n/a | n/a | n/a | n/a | n/a | n/a |
| 53 | L | n/a | n/a | n/a | n/a | n/a | n/a | n/a |
| 53 | R | n/a | n/a | n/a | n/a | n/a | n/a | n/a |
| 53 | R | n/a | n/a | n/a | n/a | n/a | n/a | n/a |
| 53 | R | n/a | n/a | n/a | n/a | n/a | n/a | n/a |
| 53 | avg. left | n/a | n/a | n/a | n/a | n/a | n/a | n/a |
| 53 | avg. right | n/a | n/a | n/a | n/a | n/a | n/a | n/a |
| 54 | L | n/a | n/a | n/a | n/a | n/a | n/a | n/a |
| 54 | L | n/a | n/a | n/a | n/a | n/a | n/a | n/a |
| 54 | L | n/a | n/a | n/a | n/a | n/a | n/a | n/a |
| 54 | R | n/a | n/a | n/a | n/a | n/a | n/a | n/a |
| 54 | R | n/a | n/a | n/a | n/a | n/a | n/a | n/a |
| 54 | R | n/a | n/a | n/a | n/a | n/a | n/a | n/a |
| 54 | avg. left | n/a | n/a | n/a | n/a | n/a | n/a | n/a |
| 54 | avg. right | n/a | n/a | n/a | n/a | n/a | n/a | n/a |
| 55 | L | n/a | n/a | n/a | n/a | n/a | n/a | n/a |
| 55 | L | n/a | n/a | n/a | n/a | n/a | n/a | n/a |
| 55 | L | n/a | n/a | n/a | n/a | n/a | n/a | n/a |
| 55 | R | n/a | n/a | n/a | n/a | n/a | n/a | n/a |
| 55 | R | n/a | n/a | n/a | n/a | n/a | n/a | n/a |
| 55 | R | n/a | n/a | n/a | n/a | n/a | n/a | n/a |
| 55 | avg. left | n/a | n/a | n/a | n/a | n/a | n/a | n/a |
| 55 | avg. right | n/a | n/a | n/a | n/a | n/a | n/a | n/a |
| 56 | L | n/a | n/a | n/a | n/a | n/a | n/a | n/a |
| 56 | L | n/a | n/a | n/a | n/a | n/a | n/a | n/a |
| 56 | L | n/a | n/a | n/a | n/a | n/a | n/a | n/a |
| 56 | R | n/a | n/a | n/a | n/a | n/a | n/a | n/a |
| 56 | R | n/a | n/a | n/a | n/a | n/a | n/a | n/a |
| 56 | R | n/a | n/a | n/a | n/a | n/a | n/a | n/a |
| 56 | avg. left | n/a | n/a | n/a | n/a | n/a | n/a | n/a |
| 56 | avg. right | n/a | n/a | n/a | n/a | n/a | n/a | n/a |
