# Supplementary material for: Prevalence and anatomical significance of the persistent median artery: A cadaveric study
Source: PLoS One. 2025 Mar 31;20(3):e0320288. doi: 10.1371/journal.pone.0320288 (PMC11957254; doi:10.1371/journal.pone.0320288)
Supplement: Attachment — Submitted filename: Response to Reviewers.docx [file pone.0320288.s004.docx]

1. I went through and revised the style of the paper to match the provided templates.
2. I added all the raw data used in this study as “Supplemental Table 3”, keeping all data anonymous/de-identified. Specimens 52-56 were immeasurable d/t preservation, AV fistulae, or other reasons; all data from these specimens is in the table as “n/a” and was not included in the calculations. Data used in **figure 2** is present in the figure legend, and therefore a second table including this information was not provided.
3. Citations in the reference list were updated and revised. An issue with the citation manager was fixed.

Add’l editor comments

1. The absence of clinical information, medical history, and knowledge of past trauma was discussed in the “Materials and Methods” section.
2. An anatomic image with labels demonstrating the median nerve, persistent median artery, and other relevant structures was included. The figure legend discusses the labels for each structure; the most important being the palmar-type persistent median artery. This image was updated with increased structure markers and better labeling.

Thank you for your time reviewing this manuscript.
